# Comparative performance of radiomics models based on 3D-FLAIR-CUBE and MPRAGE sequences gadolinium-induced labyrinth MRI protocols for predicting endolymphatic hydrops in Ménière’s disease

**DOI:** 10.1186/s40001-025-03720-y

**Published:** 2026-01-03

**Authors:** Tian Zheng, Wen Xie, Shuhao Li, Yun Peng, Jiali Liu, Xiaoping Tang, Ting Shu

**Affiliations:** 1 Department of Radiology, The Second Affiliated Hospital, Jiangxi Medical College, Nanchang University, Minde Road No. 1, Nanchang, 330006 Jiangxi Province China; 2 Jiangxi Provincial Key Laboratory of Intelligent Medical Imaging, Nanchang, 330006 Jiangxi Province China; 3 Department of Otolaryngology, The Second Affiliated Hospital, Jiangxi Medical College, Nanchang University, Minde Road No. 1, Nanchang, 330006 Jiangxi Province China

**Keywords:** Meniere's disease, Endolymphatic hydrops, Radiomics, Gadolinium-induced labyrinth MRI, Machine learning

## Abstract

**Purpose:**

This study aimed to develop and validate a radiomics-based machine learning model using 3D-FLAIR-CUBE and 3D MPRAGE gadolinium-induced labyrinth MRI protocols and accurately predict endolymphatic hydrops in unilateral Meniere’s disease.

**Methods:**

This retrospective study enrolled 175 patients with definite unilateral MD (2019–2023) who underwent three-dimensional cube liquid attenuation inversion recovery (3D-FLAIR-CUBE) and three-dimensional magnetization-prepared rapid gradient echo (3D-MPRAGE) MRI after intratympanic gadolinium injection. Radiomic features (n = 1666) from cochlear and vestibular regions were extracted using PyRadiomics, followed by feature selection the least absolute shrinkage and selection operator, (LASSO) regression and Pearson correlation. Three machine learning models logistic regression (LR), Naive Bayes (NB), and multilayer perceptron (MLP) were trained (80% data set) and tested (20%) to classify EH. Model performance was evaluated by AUC, accuracy, calibration curves and decision curve analysis (DCA).

**Results:**

In the modeling based on 3D-FLAIR-CUBE radiomic features, the machine learning models demonstrated strong capability in identifying the affected ear. The multilayer perceptron (MLP) model achieved an AUC of 0.914 in the training set and 0.815 in the test set. Analysis based on 3D-MPRAGE features yielded slightly lower yet still significant performance, with AUCs of 0.778 in the training set and 0.712 in the validation set. A combined model integrating features from both sequences showed AUCs of 0.913 and 0.814 for the training and test sets, respectively. However, DeLong’s test indicated that the combined model did not provide a statistically significant improvement compared to the model using the CUBE sequence alone. Notably, the model based on 3D-FLAIR-CUBE outperformed that based on 3D-MPRAGE. Decision curve analysis further confirmed its favorable clinical net benefit. Importantly, feature selection revealed that vestibular features carried greater diagnostic weight than cochlear features in the final model, suggesting their higher relevance in identifying endolymphatic hydrops.

**Conclusions:**

This study developed the first radiomics model based on gadolinium-induced labyrinth MRI using 3D-FLAIR-CUBE and 3D-MPRAGE sequences. The MLP model based on 3D-FLAIR-CUBE demonstrated the best diagnostic performance. Focusing on vestibular imaging features can improve the detection of endolymphatic hydrops. This tool may assist in guiding personalized treatment decisions, particularly for patients with refractory Ménière’s disease.

## Introduction

Meniere's disease (MD) is a syndrome of unknown etiology, characterized by episodic vertigo, tinnitus, aural fullness and fluctuating hearing loss [1]. In 1861, this disease was first reported by Meniere, who revealed that the lesions of the inner ear (membranous labyrinth) might be the pathological basis of MD. In 1980, Fraysse, et al. further found the presence of endolymphatic hydrops (EHs) in the autopsies of MD patients [2]. Unlike other inner ear diseases causing vertigo, EH is generally considered the hallmark of MD [3]. However, it is not easy to visualize EH directly by conventional MRI examination. Thus, the diagnostic criterion of MD proposed by the latest guideline revised in 2015 was based on patients’ symptoms and hearing test results [4]. However, given the subjectivity of clinical symptoms and the non-specific results of hearing tests, misdiagnosis still possible exists. In addition, although the symptoms of most MD patients can be improved by medical treatments, for some patients, an intratympanic injection of gentamicin is recommended due to ineffective conservative treatment [5]. Therefore, it is crucial to identify the affected ear side and degree of EH in patients with suspected MD, which is beneficial for precise diagnosis and therapeutic strategy-making.

To visualize the EH among MD patients, gadolinium-induced labyrinth MRI has been used worldwide [6]. This technology is based on the selective transport characteristics of different substances of the biological barrier of round window and oval membrane. The gadolinium contrast agent can selectively penetrate the perilymphatic space, consequently leads to preferential enhancement of the perilymph. For MD patients, images can show distended low-signal endolymphatic space caused by EH [7]. Currently, the commonly used sequences for EH visualization are 3D-fluid attenuated inversion recovery (3D-FLAIR) and 3D MPRAGE, especially the 3D-FLAIR sequence. The images of this sequence can display perilymphatic and endolymphatic space clearly, as well as EH among MD patients [7]. As for the MPRAGE sequence, although it is rarely used in displaying perilymphatic and endolymphatic space, it remains the most commonly used 3D T1weighted post-contrast sequence in clinical neuroimage [8, 9]. Due to its high isotropic resolution, 3D T1-MPRAGE provides exquisite 3D visualization to enhance the pathology of inner ear diseases [10, 11]. Regarding the EH grading methods, some researchers proposed different methods to evaluate the degree of EH, including the vestibular-based visual quartering method recommended by Bermaerts [12] and the method combining the degree of deformation of the Reissner membrane and the area ratio of scala media and scala vestibuli reported by Nakashima et al. [6]. However, the clinical value of these methods in EH assessment is controversial [13, 14]. The potential reason for this controversy is that these methods are subjective visual assessment or semiquantitative analysis, which cannot fully reflect the pathological characteristics of the lesions, and its accuracy is prone to be influenced by the radiologist’s experience.

Radiomics has been a hot topic and focus of medical research in recent years. It was first proposed by Dutch scholars Lambin et al. [15]. The principle of radiomics is that the computer extracts a large amount of image information of high-throughput medical images, performs image segmentation, feature extraction and model construction, quantify image heterogeneity caused by changes that cannot be identified by human, and then analyses specific information through deep data mining and predictive analysis [15], consequently to achieve auxiliary diagnosis, identification or prognosis prediction [16]. New data can be predicted by classical machine learning methods using known patterns, which is conducive to discovering difficult-to-identify patterns from complex combinations of multiple clinical markers [17]. Currently, the most commonly used ML method in the medical field is supervised learning. Relevant techniques include Logistic Regression (LR), Naïve Bayes (NB), decision trees, and neural networks. Among all these methods, LR is the most frequently used method of developing predictive models for dichotomous outcomes [18]. Artificial neural networks (ANN) are information processing algorithms that imitate the structure and function of brain neural networks. ANN have strong nonlinear function approximation capabilities, as well as self-organization and self-learning capabilities [19]. Coincidently, the MRI features of MD patients have strong nonlinear connections, which are suitable for analysis using ANN models. On the other hand, multilayer perceptron (MLP) neural networks have a wide range of applications and strong scalability. They use general function approximation methods to fit complex functions and can also be used to solve nonlinear classification problems.

Until now, only in some studies radiometric methods have been used to identify and evaluate EH among MD patients. Van der Lubbe [20] extracted the radiomic features of the membranous labyrinth of MD patients and healthy control individuals who underwent conventional craniocerebral T2WI and constructed a multilayer perception model to assist in diagnosing MD. However, the slice thickness was thick, the FOV was large in conventional craniocerebral T2WI, the visualization of small structures such as the vestibule and cochlea is not precision. Moreover, Chen et al. [21] conducted the radiomics analysis on 156 bilateral MD patients (312 ears) using delayed enhancement MRI with 3D-SPACE sequences. This study introduced a novel radiomic nomogram based on T2-SPACE images, which could accurately predict EH in MD patients. However, this study used only intravenous contrast delay enhancements can, double contrast increased the risk of nephrotoxicity, and single-sequence imaging provided limited information. As mentioned above, all these studies, indicated that the radiomic features could reflect the membranous labyrinth alteration, which could not be detected by subjective visual assessment, thus providing auxiliary diagnostic information for diagnosing MD, but also had some limitation.

In this study, we aims to construct a radiomic model based on 3D-FLAIR-CUBE and 3D MPRAGE gadolinium-induced labyrinth MRI scan, as well as to evaluate the diagnostic efficiency of this radiomic model in predicting the occurrence of EH in unilateral MD patients. The aim of this study is establishing an effective auxiliary diagnostic tool for MD patients, especially those who are ineffective to conservative treatment.

## Methods

### Participant

We enrolled unilateral MD patients referred to our hospital between January 2019 and February 2023. All patients had a diagnosis of definite unilateral MD. The diagnostic criteria were based on the latest guidelines revised in 2016 [22]. All patients underwent multimodal gadolinium-induced labyrinth MRI scans. The exclusion criteria were as follows: (1) images lack of any of the these MRI sequences: (1) 3D-FLAIR-CUBE or 3D-MPRAGE; (2) bilateral MD; (3) image artifacts which may affect the accuracy of diagnosis; (4) middle ear diseases, acoustic neuroma, ear trauma, barotrauma, large vestibular aqueduct syndrome, or other congenital cochlear malformations. (5) Other diseases which can independently explain the clinical symptoms of vertigo or hearing loss, including transient ischemic attack, sudden hearing loss, otosclerosis, vestibular migraine, and benign paroxysmal positional vertigo, etc.

**Fig. 1:**
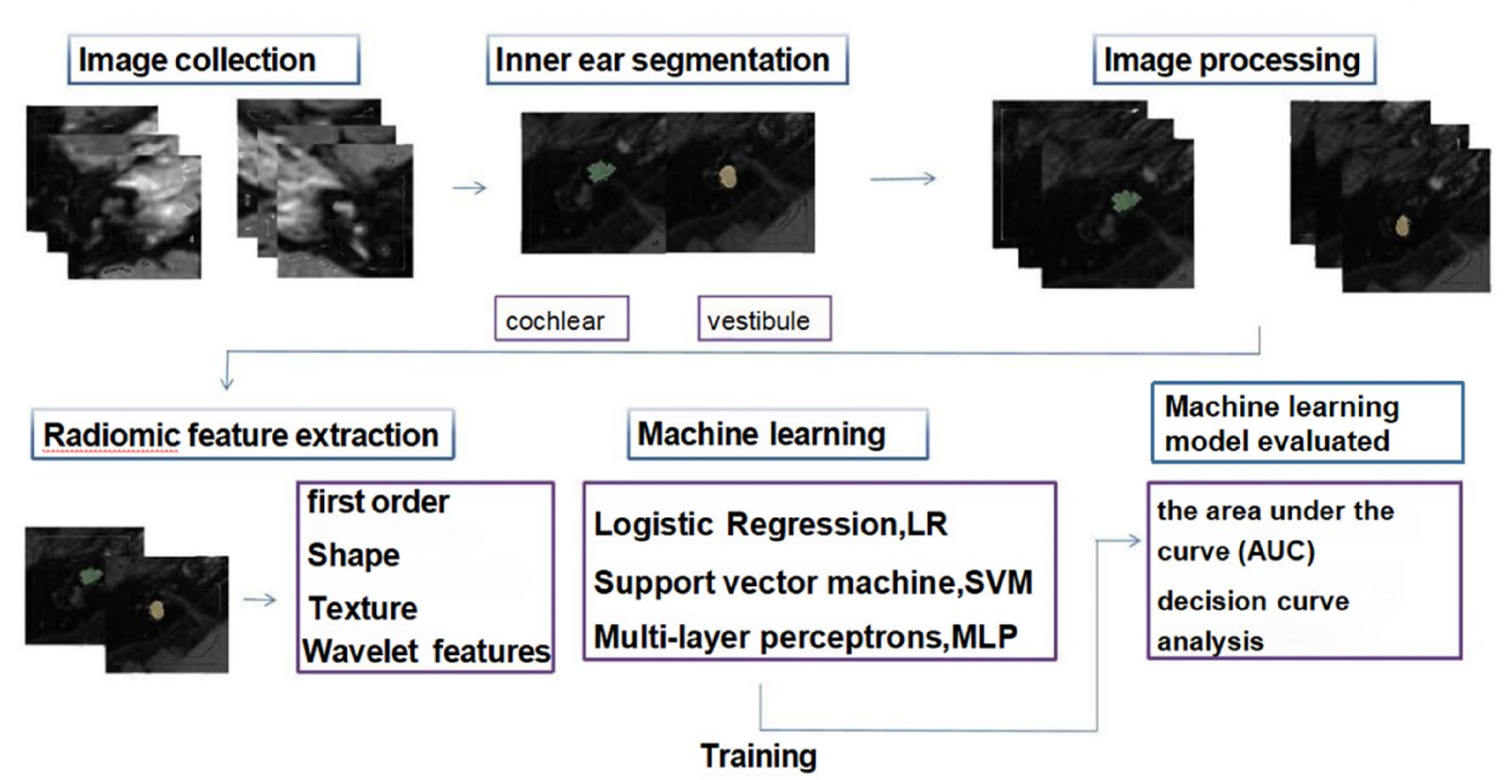
Flowchart of the study

A total of 175 patients (350 ears) were ultimately included in the analysis, comprising 175 ears with unilateral Ménière's disease that were definitively diagnosed according to established guidelines. The contralateral healthy ears of the same patients served as the control group (also 175 ears). The clinical data of these patients, including age and gender were collected. The detailed procedure is shown in Fig. 1. Images were evaluated independently by two radiologists who were blind to the patient’s clinical data. Ears with EH were divided into the positive group, and the non-EH ears were classified into the control group. All ears were randomized into the training set and test set at a ratio of 8:2.

### MRI scanning sequence and parameters

The patients’ images were carried out on a Signa 3.0T MR scanner (HDX 3.0T MR, General Electric, USA) with a 16-channel head coil. Intratympanic injection of 0.5 ml gadopentetate glucosamine diluted 1:8 with physiological saline was performed 24 h before the MRI examination. Afterward, a multimodal gadolinium-induced labyrinth MRI scan was carried out for all patients. The scan sequences included 3D-Fiesta, 3D-FLAIR-CUBE and 3D-MPRAGE, and the imaging parameters are listed in Table 1.

**Table 1:** Parameters of magnetic resonance imaging scan

	3D-FLAIR-CUBE	MPRAGE	Fiesta
Repetition time ('TR)	6000	11.4	4.6
Echo time (TE)	124.9	Min	Min
Inversion time (Tl)	1865	–	–
Flip angle (FA)	Variable, min: 20°, max120°	15	60
Echo train length (ETL)	140	–	–
Field of view (FOV)	24	20	16
Slice thickness	1.2	1.2	1.4
Gap	0	0	0
Scan time	5′39	4′46	2′49
Bandwidth	31.25	15.63	62.5

### The region of interest (ROI) sketch and feature extraction

All the images were exported as DICOM and resampled to 1, 1, or 1 pixel. An experienced radiologist who was blind to patients’ clinical information sketched the vestibule and cochlea on the 3D-FLAIR-CUBE and 3D MPRAGE images of each ear. This operation was done using the software 3D-ITKSNAP (version 4.0.1). Then, the PyRadiomics package of Python was used to extract the radiomic features of each ROI, including 324 first-order features, shape features, texture features, and wavelet features, for a total of 833 features. 1666 3D-FLAIR-CUBE and 3D MPRAGE features were obtained from all ears (833 vestibules and 833 cochlears).

### Feature screening and model construction

After normalizing all the radiomic features to the range of 0–1, we sequentially used 3D-FLAIR-CUBE and 3D MPRAGE to screen and model the features of the sequences.

Takes the 3D-FLAIR-CUBE sequence feature, for example, the feature screening process was as follows: first, Pearson correlation analysis was used to exclude strongly correlated features, and for every two features with *r* > 0.9, one of the features was randomly excluded. Second, the Lasso algorithm was used to screen the features to obtain features further with nonzero coefficients. For the remaining features, logistic regression (LR), NaiveBaye (NB), and multi-layer perceptrons (MLP) machine learning methods were used sequentially to predict the diseased or healthy ear.

### Statistics and model evaluation

All patients’ clinical characteristics, including age and gender of the training set and test set, were compared via independent samples Student's *t* test and chi-square test, respectively. The performance of each machine learning model was evaluated using the area under the curve (AUC) under the receiver operating characteristic curve (ROC). Calibration curves (CC) were employed to validate the accuracy of the model’s predicted probabilities. The clinical value of the model was further evaluated via decision curve analysis (DCA). The above radiomics analysis was performed with Python and R languages.

### Ethical approval

The study was approved by the institutional and governmental ethics committee of The Second Affiliated Hospital of Nanchang University. The requirement for individual consent was waived due to the retrospective nature of the analysis.

## Results

### Clinical characteristics

A total of 175 patients (350 ears) were ultimately included in the analysis. Among all patients, 71 were male, and 104 were female. The mean age of the patients was 56.5 years. The clinical characteristics of all patients and the MRI results of all ears in the training set and test set groups are shown in Table 2. There was no significant difference in the age and gender of patients between the two groups.

**Table 2:** Comparison of clinical characteristics and MRI results between the training set and the test set

	Age (years)	*P* value	Gender		*P* value	Lymphatic hydrops		*P* value
			Male	Female		Positive	Negative	
Training set (*n* = 280)	56.55 ± 13.56	0.069^#^	169 (60.4%)	111 (39.6%)	0.479^&^	142 (50.7%)	138 (49.3%)	0.748^#^
Test set (*n* = 70)	53.16 ± 14.27		39 (55.7)	31 (44%)		34 (48.6%**)**	36 (51.4%**)**	

^#^Student's *t* test, ^&^chi-square test

### Feature dimension reduction

**Fig. 2:**
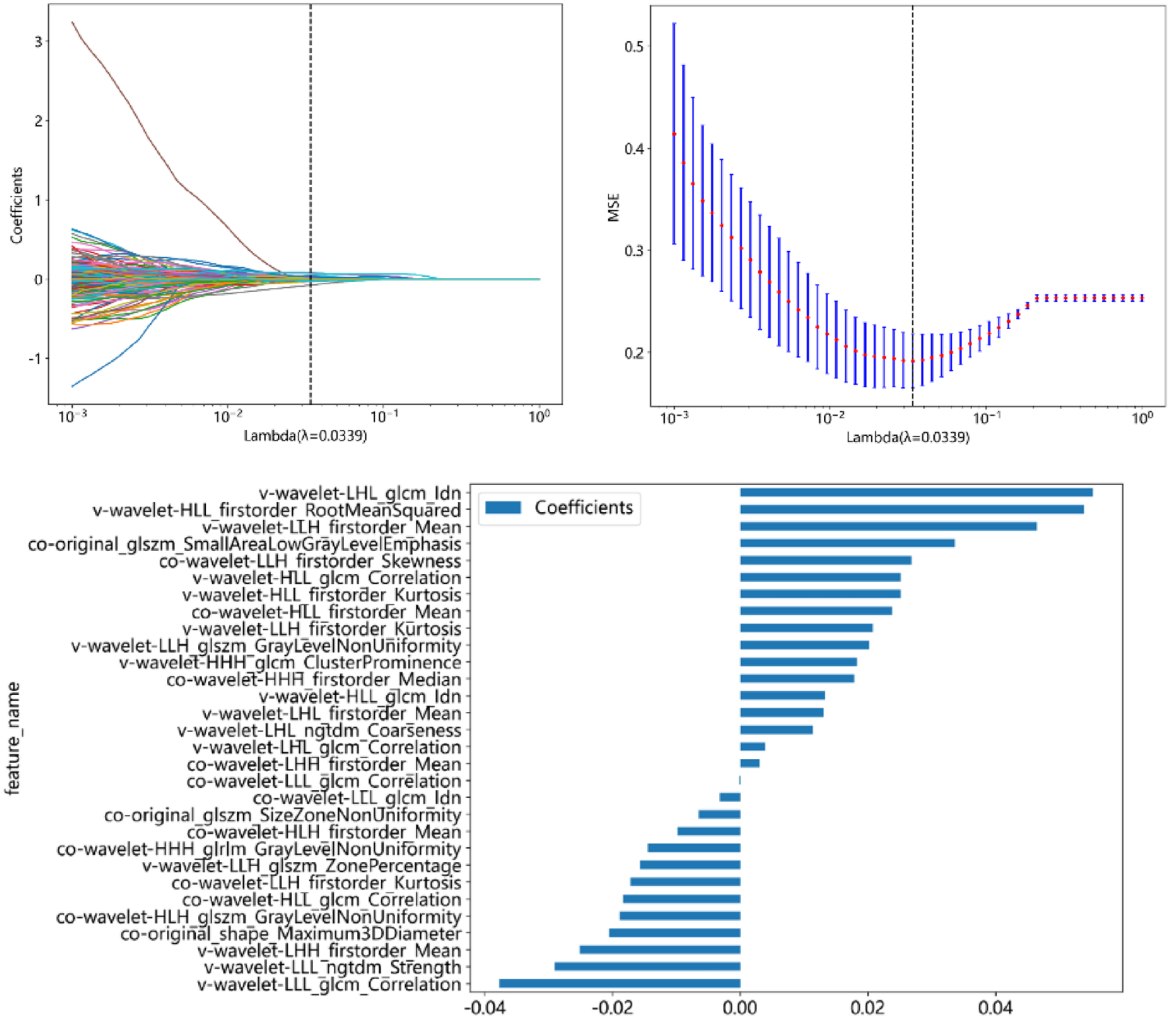
3D-FLAIR-CUBE feature selection via LASSO. **a** LASSO coefficient of the total 751 features. **b** Feature selection in LASSO based on minimum criteria. c Remaining features and their corresponding weights

For the 3D-CUBE image, 371 features were retained after Pearson correlation analysis. The Lasso algorithm further selected 29 features with nonzero coefficients (Fig. 2), of which 15 were cochlear radiomics features, including Co-original_glszm_SmallAreaLowGrayLevelEmphasis, Co-original_shape_Maximum3DDiameter, Co-wavelet-HHH_glrlm_GrayLevelNonUniformity, Co-wavelet-HHL_glszm_GrayLevelNonUniformity, Co-wavelet-HLH_firstorder_Mean, Co-wavelet-HLH_glcm_Correlation, Co-wavelet-HLH_glszm_ZoneVariance, Co-wavelet-HLL_glcm_Correlation, Co-wavelet-LHH_firstorder_Mean, Co-wavelet-LHH_firstorder_Skewness, Co-wavelet-LHH_glcm_ClusterShade, Co-wavelet-LLH_firstorder_Kurtosis, Co-wavelet-LLH_firstorder_Skewness, Co-wavelet-LLH_glcm_Correlation, Co-wavelet-LLL_glcm_Correlation, Ve-wavelet-HLL_firstorder_RootMeanSquared. There were 14 vestibular features, including Ve-wavelet-HLL_firstorder_Skewness, Ve- wavelet-HLL_glcm_Correlation, Ve-wavelet-HLL_glcm_Idn, Ve-wavelet-LHH_firstorder_Median, Ve-wavelet-LHL_firstorder_Mean, Ve-wavelet-LHL_glcm_Correlation, Ve-wavelet-LHL_glcm_Idn, Ve-wavelet-LHL_glszm_ZonePercentage, Ve-wavelet-LHL_ngtdm_Coarseness, Ve-wavelet-LLH_firstorder_Kurtosis, Ve-wavelet-LLH_firstorder_Mean, Ve-wavelet-LLH_glszm_GrayLevelNonUniformity, and Ve-wavelet-LLL_ngtdm_Strength.

**Fig. 3:**
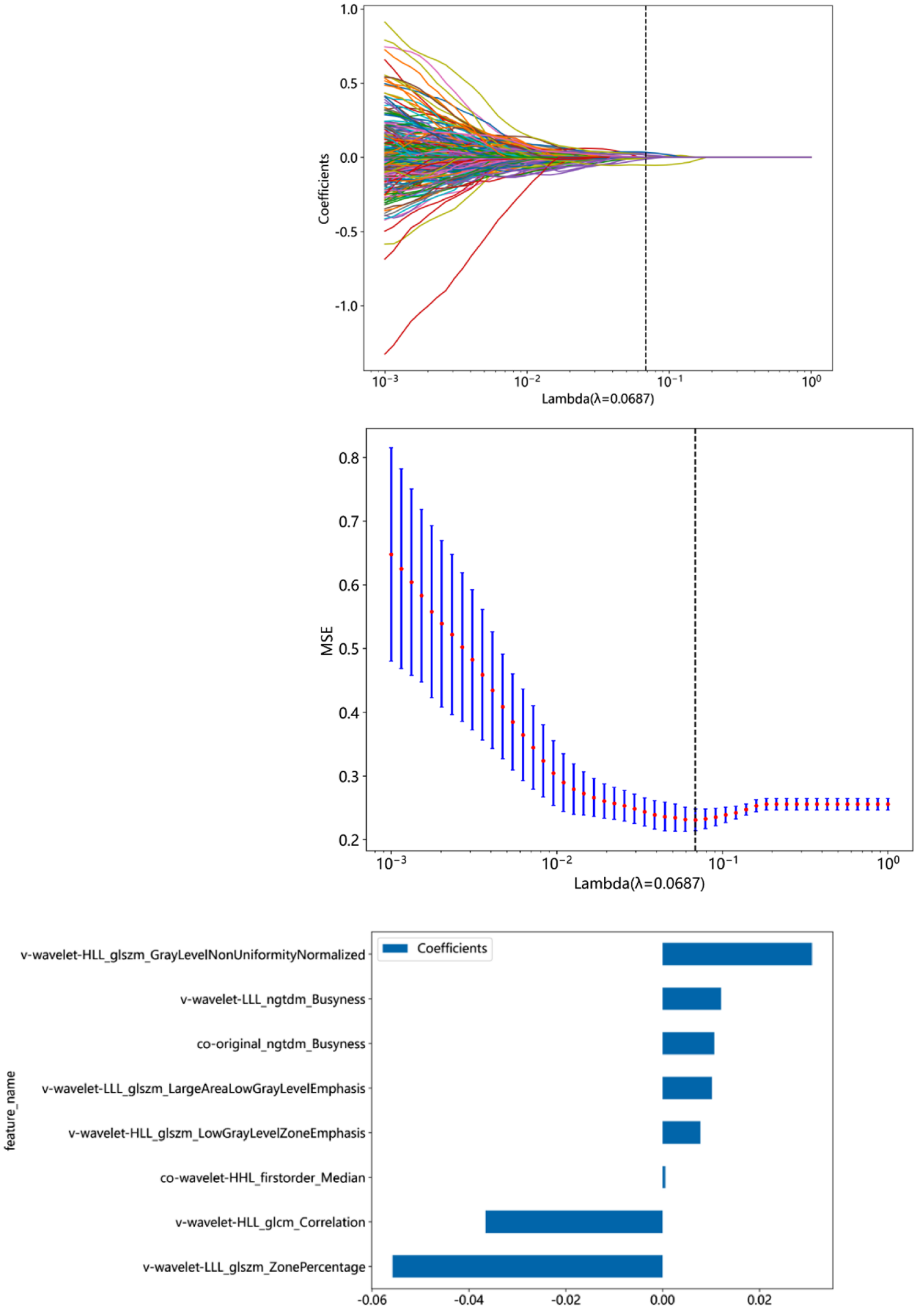
3D MPRAGE feature selection via LASSO. **a** LASSO coefficient of the total 506 features. **b** Feature selection in the LASSO model using tenfold cross-validation based on minimum criteria. **c** Remaining features and their corresponding weights

For the MPRAGE image features, 506 features remained after Pearson correlation analysis. The Lasso algorithm further selected 8 features with nonzero coefficients (Fig. 3), of which 2 were cochlear radiomic features, including Co-original_ngtdm_Busyness and Co-wavelet-HHL_firstorder_Median. There were 6 vestibular features, including Ve-wavelet-HLL_glcm_Correlation, Ve-wavelet-HLL_glszm_GrayLevelNonUniformityNormalized, Ve-wavelet-HLL_glszm_LowGrayLevelZoneEmphasis, Ve-wavelet-LLL_glszm_LargeAreaLowGrayLevelEmphasis Ve-wavelet-LLL_glszm_ZonePercentageVe-wavelet-LLL_ngtdm_Busyness.

### Model efficacy evaluation

**Fig. 4:**
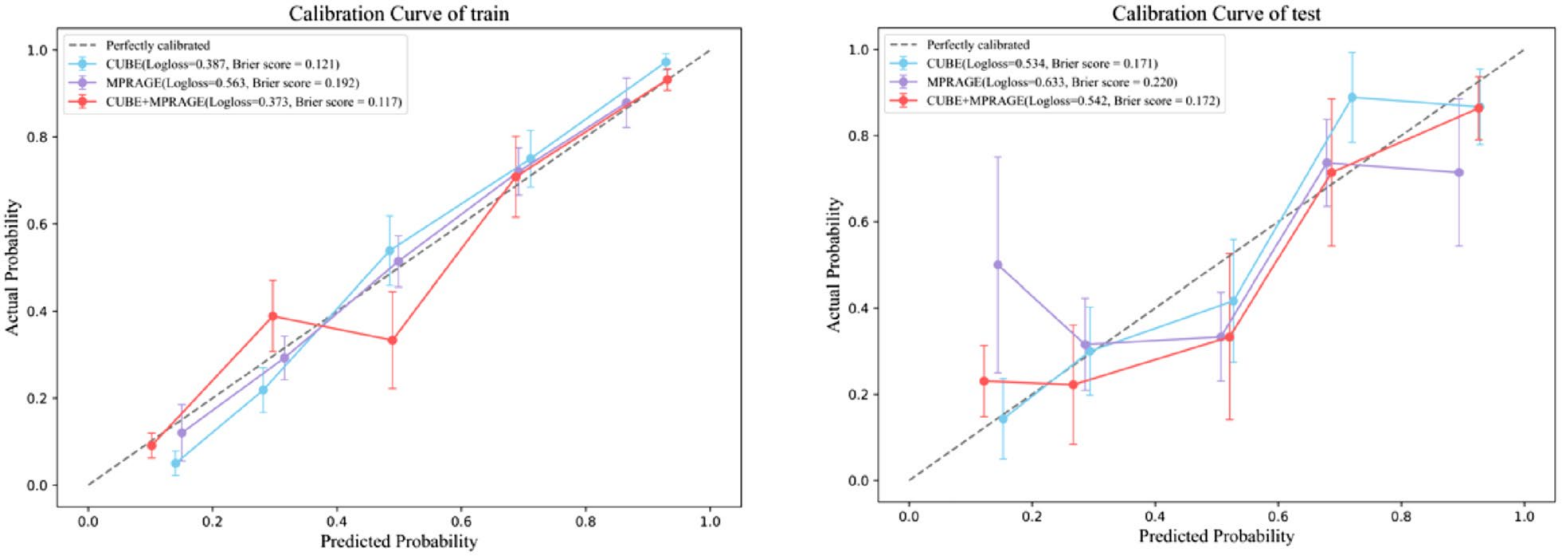
Calibration curve between the predicted probabilities of the CUBE‑based MLP model and the actual outcomes

The AUCs of the 3D-FLAIR-CUBE radiomics features for the LR, NB and MLP models on the training set were 0.875, 0.835, and 0.914, respectively. The AUCs of the test set were 0.794, 0.792, and 0.815, respectively, as shown in Table 3. In terms of 3D MPRAGE, The AUCs of LR, NB, and MLP on the training set were 0.763, 0.757, and 0.778, respectively; the AUCs of the test set were 0.699, 0.673, and 0.712, respectively (Table 4). Based on the analysis above, the MLP model based on 3D-FLAIR-CUBE demonstrates high accuracy in distinguishing between ears with EH and non-EH (Fig. 4).

**Table 3:** Performance of machine learning models based on 3D-FLAIR-CUBE radiomics features in identifying affected and unaffected ears

Model name	Task	Accuracy	AUC	95% CI	SPE	SEN	PPV	NPV
LR	Train	0.811	0.875	0.8343–0.9161	0.768	0.855	0.845	0.781
	Test	0.771	0.794	0.6858–0.9024	0.676	0.861	0.821	0.738
Naive Bayes	Train	0.782	0.835	0.7875–0.8827	0.746	0.819	0.809	0.758
	Test	0.771	0.792	0.6776–0.9057	0.706	0.833	0.8	0.75
MLP	Train	0.843	0.914	0.8815–0.9471	0.796	0.891	0.883	0.809
	Test	0.786	0.815	0.7107–0.9200	0.676	0.889	0.852	0.744

**Table 4:** Performance of machine learning models based on 3D MPRAGE radiomics features in identifying affected and unaffected ears

Model name	Task	Accuracy	AUC	95% CI	SPE	SEN	PPV	NPV
LR	Train	0.704	0.763	0.7077–0.8184	0.711	0.696	0.706	0.701
	Test	0.7	0.699	0.5718–0.8253	0.471	0.917	0.842	0.647
Naive Bayes	Train	0.689	0.757	0.7012–0.8122	0.627	0.754	0.724	0.662
	Test	0.671	0.673	05435–0.8029	0.559	0.778	0.704	0.651
MLP	Train	0.718	0.778	0.7243–0.8315	0.641	0.797	0.765	0.683
	Test	0.714	0.712	0.5858–0.8374	0.529	0.889	0.818	0.667

**Fig. 5:**
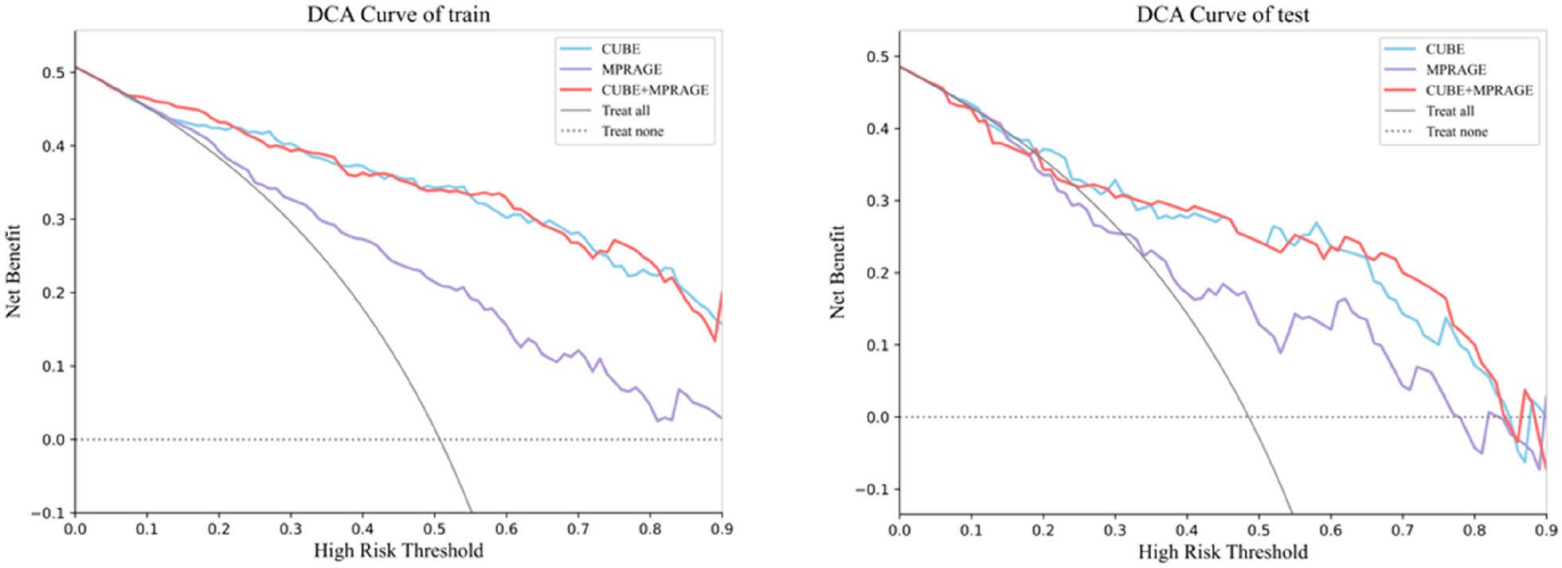
Decision curve analysis of the MLP model based on 3D-FLAIR-CUBE, 3D MPRAGE and combined model

A combined model integrating features from both sequences was developed. The AUC values for the training and test sets of this model were (0.913, 0.814), respectively. However, DeLong’s test indicated that the combined model did not yield a statistically significant improvement compared to the model based solely on the CUBE sequence. The calibration curve (Fig. 4) demonstrated good agreement between the predicted probabilities of the CUBE‑based MLP model and the actual outcomes. Decision curve analysis (Fig. 5) further revealed that, across threshold ranges of 12–100% (0–6%) in the training set and 15–85% (0–11%) in the test set, the CUBE model provided an equal to or higher net clinical benefit than the MPRAGE model (Table 5).

**Table 5:** Model evaluation results of training and test set based on 3D CUBE, 3D MPRAG and combined model

Model name	Task	Accuracy	AUC	95% CI	SPE	SEN	PPV	NPV	*P*^#^
CUBE	Train	0.846	0.914	0.881–0.947	0.891	0.803	0.884	0.815	0
	Test	0.8	0.815	0.711–0.920	0.889	0.706	0.857	0.762	0
MPRAGE	Train	0.721	0.778	0.724–0.831	0.797	0.648	0.767	0.688	–0.14
	Test	0.729	0.712	0.586–0.837	0.889	0.559	0.826	0.681	–0.1
CUBE + MPRAGE	Train	0.85	0.913	0.880–0.946	0.899	0.803	0.891	0.816	0
	Test	0.8	0.814	0.709–0.919	0.889	0.706	0.857	0.762	0

^#^*P* values of Delong test between two models

## Discussion

In this study, we extracted the radiomics features of the cochlea and vestibule of MD patients using the 3D-FLAIR-CUBE and 3D MPRAGE sequences, and constructed the LR, NB and MLP models to predict EH in unilateral MD patients. We found that the model can reach a moderate to good performance in identifying EH. After screening the radiomics features, we found the weights of the remaining vestibular features were greater than those of the cochlear features. In addition, the performance of the models based on 3D-FLAIR-CUBE was generally better than 3D MPRAGE, and the MLP model outperforms other models. In this study, the AUC of the training set and test set using the 3D-FLAIR-CUBE MLR model were 0.914 and 0.815, respectively. The MLP model can effectively and accurately predict EH.

This study is the first report applying a machine learning model based on 3D-FLAIR-CUBE and 3D MPRAGE gadolinium-induced labyrinth MRI results to identify the EH among patients with unilateral MD. There are some previous studies focusing on the radiomic features of MD patients using other MRI technologies. In 2016, van den Burg [23] first reported a study based on high-resolution T2-weighted MRI scans of the labyrinth, and several differences were found in image features between the MD group and the control group using a quantitative radiomics approach. In 2021, van der Lubbe et al. [20] conducted a multicenter study which further extracted the radiomic features of conventional T2W images of MD patients and healthy control individuals. They found that using the MLP classification model could achieved a precise, high-diagnostic performance in identifying MD patients. However, these studies have some limitations. For instance, the EH assessment methods used in these studies were conventional inner ear sequences rather than enhancement MRI. In a recent study, Chen [21] demonstrated that the novel radiomics nomograms based on enhanced T2-SPACE images were successfully constructed to predict cochlear and vestibular EH in MD patients. Although previous studies have explored the value of applying radiomics to MD patients, this study is different from these studies in two aspects. First, we extracted the radiomics features of both cochlea and vestibule, and constructed a machine-learning model on the basis of the features extracted from these two structures. We found that the vestibular features played a more significant role in predicting EH models. Second, instead of using a single modality, we performed radiomics analysis based on two image sequences, 3D-FLAIR-CUBE, and 3D MPRAGE, to evaluate which sequence was better in identifying EH.

With the improvement of computing power, more and more machine learning algorithms have been proposed. This study used three commonly used algorithms. Among them, MLP has the highest performance. Compared with ANN models, MLP is a typical feedforward neural network, with particular advantages in dealing with nonlinear continuous variable relationships. By contrast, the ANN model is more accurate in classifying the dependent variable than the LR model [19]. Many studies have shown it performs well in disease prediction and diagnosis [19, 20]. This study yielded similar results that is the MLP model performed better in identifying EH in MD patients.

After screening the features, we found that the vestibular features usually had a higher weight and were more dominant in number, which is consistent with previous studies. Yu et al. [24] analyzed the images of delayed enhanced MRI of patients with EH and found that EH mainly occurred in the vestibule, which was often presented as morphological abnormalities. In addition, Kahn et al. [25] evaluated the dilation of the endolymphatic space in the cochlea, saccule, utricle, and ampulla of the semicircular canal among MD patients. They found that 88% of symptomatic ears presented EH in the cochleae, and 91% had EH in the vestibule. Three possible reasons may explain the higher prevalence of vestibule EH in MD patients. The first one is the biomechanical factor, the indeed hoop stress disparities within the variously configured containment membranes (saccule, utricle, ampulla, and semicircular canal). Pender et al. [26] confirmed that under static physiological pressure, the ratio of the tensile stress level in the saccule, utricle, ampulla, and semicircular canal was 38.8:5.4:2.4:1, and an uneven stress vulnerability existed within the vestibular membranes. These characteristics of the vestibule make it easier to swell and dilate, especially the saccule. Another reason is endolymphatic regulation failure. Longitudinal flow is dominant in abnormal endolymphatic volume status [27]. The vestibule is directly adjacent to the cochlear duct and can, therefore, act as a reservoir of endolymph [28]. When the endolymphatic sac or duct is occluded, the obstruction of longitudinal blood flow first leads to endolymphatic fluid accumulation in the vestibule, making it easier to dilate than cochlea. The third reason is that the Bast's valve plays a role in the occurrence of vestibular EH. Bast's valve prevents endolymphatic fluid backflow from the utricle [28], leading to vestibular edema, especially in the posterior vestibule, which is more severe than cochlear edema. This finding suggests that in future studies, quantitative feature analysis based on vestibular shape may provide more information for EH identification and assessment.

This study found that the radiomics model based on 3D-FLAIR-CUBE MRI outperformed T1MPRAGE. However, the performance of 3D-FLAIR-CUBE and 3D T1 MPRAGE enhanced scans in detecting lesions of other brain disease is controversial in previous studies. In a study,71 patients with suspected metastatic disease underwent contrast-enhanced MRI scanning using different sequences, and the application of 3D-FLAIR-CUBE significantly improved the detection rate of brain metastases compared with MPRAGE [29]. Another study, which included 232 multiple sclerosis patients who underwent contrast-enhanced scanning, showed that small brain veins may affect the detection accuracy of enhancing lesions on 3D-FLAIR-CUBE images compared with standard MPRAGE [30]. Previous studies have mainly focused on exploring the brain MRI results using different sequences. However, studies comparing gadolinium-based membranous labyrinth imaging using various sequences are rare. The higher sensitivity of 3D-FLAIR-CUBE in detecting cochlear and vestibular EH may be due to its higher signal-to-noise ratio (SIR) [31] and its greater robustness in displaying cochlear blood–labyrinth barrier (BLB) lesions. Different flip angle evolution (possibly introduced in the form of off-resonance pulses) can cause a magnetization transfer effect. The sequence of 3D-FLAIR has a higher magnetization transfer effect than MPRAGE, which is considered to be another factor affecting enhancing lesions [32]. It preferentially reduces the signal from the brain parenchyma (especially white matter), making the enhancing lesions more prominent [29]. Our findings indicate that the MLP model derived from 3D‑FLAIR‑CUBE images demonstrated better diagnostic performance than the 3D‑MPRAGE‑based model, and a combined model did not provide added diagnostic value. Therefore, the selection of the gadolinium-induced labyrinth MRI could be individualized based on patient‑specific factors.

## Limitation

This study has several limitations. First, as a single-center retrospective investigation, it serves as a preliminary exploration of gadolinium-induced labyrinth MRI in Ménière’s disease. Although the developed models performed well, their generalizability and clinical applicability require further validation through multicenter or prospective cohort studies. Second, the present study did not adopt a two‑independent‑reader approach for ROI delineation. While all ROIs were manually segmented by an experienced head‑and‑neck imaging specialist and reviewed by a senior radiologist, future studies would benefit from involving multiple readers and evaluating inter‑observer consistency using the intraclass correlation coefficient (ICC). Third, we did not examine the correlation between radiomic features and clinical laboratory indicators, such as disease stage, attack frequency, severity and fluctuation patterns of hearing loss, and related vestibular examinations. Finally, longitudinal data including treatment modalities and efficacy follow‑up were not incorporated. Future work will focus on addressing these directions to further improve the clinical relevance of the model.

## Conclusion

The machine learning models developed from gadolinium-induced labyrinth MRI using 3D‑FLAIR‑CUBE and 3D‑MPRAGE sequences demonstrated moderate‑to‑good performance in identifying EH in unilateral Ménière’s disease. Notably, the model based on the 3D‑FLAIR‑CUBE sequence outperformed that based on 3D‑MPRAGE. In addition, quantitative features derived from vestibular morphology may provide further valuable information for the detection and assessment of hydrops.

## Data Availability

No datasets were generated or analysed during the current study.

## References

[1] Gordon AG. Ménière’s disease. Lancet. 2006. 10.1016/S0140-6736(06)68419-5.16631897

[2] Bernard G, Fraysse MDT, Alomso A, House WF. Meniere's disease and endilymphatic hydrops clinical-hiistopathological correlations. Meniere's disease and endolymphatic hydrops. 1980. 10.1177/00034894800896s201 6779694

[3] van Steekelenburg JM, et al. Value of endolymphatic hydrops and perilymph signal intensity in suspected Ménière disease. AJNR Am J Neuroradiol. 2020;41(3):529–34. 32029469 10.3174/ajnr.A6410 PMC7077918

[4] Lopez-Escamez JA, et al. Diagnostic criteria for Menière’s disease. J Vestib Res. 2015;25(1):1–7. 25882471 10.3233/VES-150549

[5] Nevoux J, et al. International consensus (ICON) on treatment of Ménière’s disease. Eur Ann Otorhinolaryngol Head Neck Dis. 2018;135(1):S29–32. 29338942 10.1016/j.anorl.2017.12.006

[6] Nakashima T, et al. Grading of endolymphatic hydrops using magnetic resonance imaging. Acta Otolaryngol. 2009;129(sup560):5–8. 10.1080/00016480902729827 19221900

[7] Naganawa S, et al. Three-dimensional (3D) visualization of endolymphatic hydrops after intratympanic injection of Gd-DTPA: optimization of a 3D-real inversion-recovery turbo spin-echo (TSE) sequence and application of a 32-channel head coil at 3T. J Magn Reson Imaging. 2009;31(1):210–4. 10.1002/jmri.22012 20027590

[8] Ellingson BM, et al. Consensus recommendations for a standardized brain tumor imaging protocol for clinical trials in brain metastases. Neuro Oncol. 2020;22(6):757–72. 32048719 10.1093/neuonc/noaa030 PMC7283031

[9] Traboulsee A, et al. Revised recommendations of the Consortium of MS Centers Task Force for a standardized MRI protocol and clinical guidelines for the diagnosis and follow-up of multiple sclerosis. AJNR Am J Neuroradiol. 2016;37(3):394–401. 26564433 10.3174/ajnr.A4539 PMC5094650

[10] Ngamsombat. ALMGFKMAJCC, Stephen KS, Cauley F, Liu W, Splitthoff DN, Lo W-C, et al. Validation of a highly accelerated post-contrast wave-controlled aliasing in parallel imaging (CAIPI) 3D-T1 MPRAGE compared to standard 3D-T1 MPRAGE for detection of intracranial enhancing lesions on 3-T MRI. Eur Radiol. 2023;33:2905–15. 36460923 10.1007/s00330-022-09265-6 PMC9718459

[11] Shah M, Ross JS, Tkach J, Modic MT. 3D MPRAGE evaluation of the internal auditory canals. J Comput Assist Tomogr. 1993;17(3):442–5. 8491908 10.1097/00004728-199305000-00020

[12] Bernaerts A, et al. The value of four stage vestibular hydrops grading and asymmetric perilymphatic enhancement in the diagnosis of Menière’s disease on MRI. Neuroradiology. 2019;61(4):421–9. 30719545 10.1007/s00234-019-02155-7 PMC6431299

[13] Xie W, et al. The relationship between clinical characteristics and magnetic resonance imaging results of Ménière disease: a prospective study. Sci Rep. 2021. 10.1038/s41598-021-86589-1. 33785791 10.1038/s41598-021-86589-1 PMC8010013

[14] Hu Y, et al. Endolymphatic hydrops imaging and correlation with clinical characteristics, audiovestibular function and mental impairment in patients with Meniere’s disease. Eur Arch Otorhinolaryngol. 2023;280(9):4027–36. 36849561 10.1007/s00405-023-07899-w PMC10382354

[15] Lambin P, et al. Radiomics: extracting more information from medical images using advanced feature analysis. Eur J Cancer. 2012;48(4):441–6. 22257792 10.1016/j.ejca.2011.11.036 PMC4533986

[16] Alaimo L, et al. Development and validation of a machine-learning model to predict early recurrence of intrahepatic cholangiocarcinoma. Ann Surg Oncol. 2023;30(9):5406–15. 37210452 10.1245/s10434-023-13636-8

[17] Jiang F, et al. Artificial intelligence in healthcare: past, present and future. Stroke Vasc Neurol. 2017;2(4):230–43. 29507784 10.1136/svn-2017-000101 PMC5829945

[18] Hsieh M-H, et al. The performance of different artificial intelligence models in predicting breast cancer among individuals having type 2 diabetes mellitus. Cancers. 2019. 10.3390/cancers11111751. 31717292 10.3390/cancers11111751 PMC6895886

[19] Renganathan V. Overview of artificial neural network models in the biomedical domain. Bratisl Med J. 2019;120(07):536–40. 10.4149/BLL_2019_087 31602991

[20] van der Lubbe MFJA, et al. A non-invasive, automated diagnosis of Menière’s disease using radiomics and machine learning on conventional magnetic resonance imaging: a multicentric, case-controlled feasibility study. Radiol Med (Torino). 2021;127(1):72–82. 34822101 10.1007/s11547-021-01425-w PMC8795017

[21] Chen W, et al. A novel radiomics nomogram based on T2-sampling perfection with application-optimized contrasts using different flip-angle evolutions (SPACE) images for predicting cochlear and vestibular endolymphatic hydrops in Meniere’s disease patients. Eur Radiol. 2024. 10.1007/s00330-024-10670-2. 38457037 10.1007/s00330-024-10670-2

[22] Goebel JA. 2015 equilibrium committee amendment to the 1995 AAO-HNS guidelines for the definition of Ménière’s disease. Otolaryngol Head Neck Surg. 2016;154(3):403–4. 26884364 10.1177/0194599816628524

[23] van den Burg EL, et al. An exploratory study to detect Ménière’s disease in conventional MRI scans using radiomics. Front Neurol. 2016;7:190. 27872606 10.3389/fneur.2016.00190 PMC5098221

[24] Yu J, et al. Different findings of morphological changes and functional decline in the vestibule and the semicircular canal in ipsilateral delayed endolymphatic hydrops. Clin Neurophysiol. 2020;131(7):1487–94. 32388473 10.1016/j.clinph.2020.03.032

[25] Kahn L, et al. Relationship between video head impulse test, ocular and cervical vestibular evoked myogenic potentials, and compartmental magnetic resonance imaging classification in menière’s disease. The Laryngoscope. 2019;130(7):E444–52. 31742710 10.1002/lary.28362

[26] Pender D. Membrane stress in the human labyrinth and Meniere disease: a model analysis. Int Arch Otorhinolaryngol. 2015;19(04):336–42. 26491481 10.1055/s-0035-1549157 PMC4593924

[27] Salt AN, Plontke SK. Endolymphatic hydrops: pathophysiology and experimental models. Otolaryngol Clin N Am. 2010;43(5):971–83. 10.1016/j.otc.2010.05.007 PMC2923478 20713237

[28] Jasińska A, et al. Correlation between magnetic resonance imaging classification of endolymphatic hydrops and clinical manifestations and audiovestibular test results in patients with definite Ménière’s disease. Auris Nasus Larynx. 2022;49(1):34–45. 33865653 10.1016/j.anl.2021.03.027

[29] Kim D, et al. Usefulness of the delay alternating with nutation for tailored excitation pulse with T1-weighted sampling perfection with application-optimized contrasts using different flip angle evolution in the detection of cerebral metastases: comparison with MPRAGE imaging. Am J Neuroradiol. 2019;40:1469–75. 31371358 10.3174/ajnr.A6158 PMC7048452

[30] Danieli L, et al. Nonlesional sources of contrast enhancement on postgadolinium “black-blood” 3D T1-SPACE images in patients with multiple sclerosis. AJNR Am J Neuroradiol. 2022;43(6):872–80. 35618421 10.3174/ajnr.A7529 PMC9172944

[31] Bernaerts A, et al. Comparison between 3D SPACE FLAIR and 3D TSE FLAIR in Menière’s disease. Neuroradiology. 2022;64(5):1011–20. 35149883 10.1007/s00234-022-02913-0 PMC9005391

[32] Constable RT, Anderson AW, Zhong J, Gore JC. Factors influencing contrast in fast spin-echo MR imaging. Magn Reson Imaging. 1992;10:497–511. 1501520 10.1016/0730-725x(92)90001-g

